# Tree species distribution in temperate forests is more influenced by soil than by climate

**DOI:** 10.1002/ece3.3436

**Published:** 2017-10-11

**Authors:** Lorenz Walthert, Eliane Seraina Meier

**Affiliations:** ^1^ Swiss Federal Institute for Forest Snow and Landscape Research WSL Birmensdorf Switzerland; ^2^ Agroscope Institute for Sustainability Sciences ISS Zurich Switzerland

**Keywords:** drought, ecological niche, gradient analysis, nutrients, soil aeration, species abundance, species distribution models, variation partitioning

## Abstract

Knowledge of the ecological requirements determining tree species distributions is a precondition for sustainable forest management. At present, the abiotic requirements and the relative importance of the different abiotic factors are still unclear for many temperate tree species. We therefore investigated the relative importance of climatic and edaphic factors for the abundance of 12 temperate tree species along environmental gradients. Our investigations are based on data from 1,075 forest stands across Switzerland including the cold‐induced tree line of all studied species and the drought‐induced range boundaries of several species. Four climatic and four edaphic predictors represented the important growth factors temperature, water supply, nutrient availability, and soil aeration. The climatic predictors were derived from the meteorological network of MeteoSwiss, and the edaphic predictors were available from soil profiles. Species cover abundances were recorded in field surveys. The explanatory power of the predictors was assessed by variation partitioning analyses with generalized linear models. For six of the 12 species, edaphic predictors were more important than climatic predictors in shaping species distribution. Over all species, abundances depended mainly on nutrient availability, followed by temperature, water supply, and soil aeration. The often co‐occurring species responded similar to these growth factors. Drought turned out to be a determinant of the lower range boundary for some species. We conclude that over all 12 studied tree species, soil properties were more important than climate variables in shaping tree species distribution. The inclusion of appropriate soil variables in species distribution models allowed to better explain species' ecological niches. Moreover, our study revealed that the ecological requirements of tree species assessed in local field studies and in experiments are valid at larger scales across Switzerland.

## 1 INTRODUCTION

Knowledge on the ecological requirements determining tree species distributions is a precondition for profitable and sustainable forest management and for forest conservation under current and future environmental conditions. In the last decades, numerous studies have been conducted to develop and to refine the methodology for assessing tree species responses to the environment. Thus, a reliable conceptual framework for assessing the ecological requirements for tree species distributions should consider mainly two methodical issues. First, an adequate number of observations (Coudun & Gegout, 2006) should be investigated along diverse and strong environmental gradients including species range boundaries (Beauregard & de Blois, 2014). However, species do not always reach their distribution edges within the investigation area of studies, and, thus, this precondition is often not fulfilled. Second, environmental factors with a direct impact on plant performance (e.g., temperature and nutrients) should be preferred to indirect factors (e.g., elevation and geology), because direct factors are ecologically more comprehensible and have a larger spatial applicability (Austin & Cunningham, 1981; Guisan & Zimmermann, 2000). However, the data available for direct factors are most often not spatially explicit, for example, biotic interactions, disturbances, and soil (Mod, Scherrer, Luoto, Guisan, & Scheiner, 2016). Thus, direct factors and, more specifically, soil characteristics are, despite their importance, rarely considered in predictions of species distributions (Diekmann, Michaelis, & Pannek, 2015; Thuiller, 2013). In cases where soil‐related information has been used in studies, it has often been derived from bioindication (e.g., Piedallu, Gegout, Lebourgeois, & Seynave, 2016) and is therefore considered to suffer from ambiguity and thus from limited comparability to measured soil variables (Szymura, Szymura, & Maciol, 2014).

Only a few studies have considered measured soil variables in species distribution models (SDM) for characterizing ecological niches (e.g., Dubuis et al., 2013; Pinto & Gegout, 2005; Walthert, Graf Pannatier, & Meier, 2013) or for predicting species distributions (e.g., Beauregard & de Blois, 2014; Coudun, Gegout, Piedallu, & Rameau, 2006; Piedallu, Gegout, Perez, & Lebourgeois, 2013). These studies have shown that the statistical model performance was better for most of the studied plant species if edaphic variables were added to climatic factors.

For many European tree species growing in the temperate zone, it is still unclear if and how they react to soil properties, as the existing studies included only a few tree species or did not fully consider the methodical requirements mentioned above; that is, observations should cover strong environmental gradients including species distribution edges, use of direct instead of indirect factors, and use of measured soil variables instead of ecological indicator values.

Climate and soil are among the most important growth factors and thus drivers of tree species distributions. Important climatic factors are temperature and water, and important edaphic factors are water, nutrients, and probably soil aeration. Thus, in our study we investigate the importance of climatic (i.e., temperature and water) and edaphic factors (i.e., water, nutrients, and soil aeration) for the cover abundance of 12 temperate European tree species (i.e., three coniferous and nine deciduous species). More specifically, our study encompasses the three following thematic fields: First, we aim at testing for which tree species the model performance can be improved by adding soil variables to climatic variables, and how much the species differ in their sensitivity to climate and soil. We expect that including soil variables improves the model performance for all species and that the common species have the lowest sensitivity to soil properties. Moreover, we want to assess the degree of redundancy between the climate and the edaphic variables in explaining species distribution. Second, we analyze the relative importance of the four growth factors (i.e., temperature, water, nutrients, and soil aeration) in shaping species abundances. We expect similar response patterns for co‐occurring species. Third, we evaluate species' sensitivity to drought and soil oxygen shortage. We expect that drought is an important factor in determining the lower range boundary of several tree species. In addition, we review whether the results of our sensitivity assessment that is based on large‐scale inventory data agree with the outcomes of corresponding research from local case studies and from experiments. As our study focuses on the relative importance of soil and climate variables, we do not include biotic interactions and disturbances.

## 2 MATERIALS AND METHODS

### 2.1 Study area

**Figure 1:**
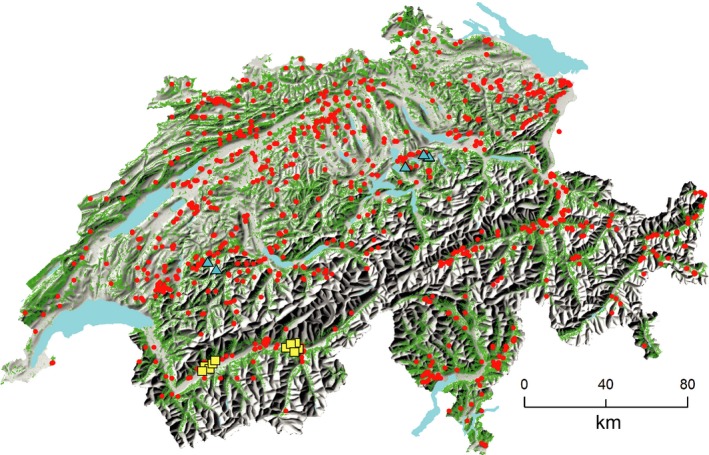
Locations of the 1,075 study plots in Switzerland. Red dots show the 1,060 mature forest stands, yellow squares the 10 treeless plots on extremely dry soils, and blue triangles the five treeless sites on marshes. Forest area is shown in green

The study area included all of Switzerland (circa 45–47°N and 6–10°E), which is located in the center of Western Europe (Fig. 1). Due to the high variability of topography, climate, and geology, Switzerland has relatively strong environmental gradients compared to its small surface. Roughly 30% of the country (12,000 km^2^) is covered with forest, half of which is located above 1,000 m a.s.l. Forest management is mainly practiced at low elevations, where no large‐scale clear‐cutting is applied and natural regeneration is often fostered by silvicultural management (Brassel & Brändli, 1999). Fertilizing or liming to stimulate soil fertility has always been forbidden in Swiss forests.

### 2.2 Study plots, species, and environmental data

#### 2.2.1 Study plots

Species and environmental data originate from a database of the Swiss Federal Institute for Forest, Snow and Landscape Research (WSL) containing data on ~1,200 mainly forested plots across Switzerland. For each plot, data from a floristic inventory conducted according to Braun‐Blanquet (Braun‐Blanquet, 1964) and soil data from a soil pit were available. For most plots, the vegetation survey and soil sampling were carried out in the same year or with a maximum time of 5 years between them. Most plots were selected according to ecological criteria for forest sites; that is, species composition and stand structure should be close to those observed in natural forests. From this dataset, we excluded about 90 forest plots according to three criteria. First, we only selected mature forest stands because we expected that the site requirements of tree species are more apparent in mature than in juvenile stands. We therefore removed all plots with juvenile forests (*n* = 32) from the dataset by excluding forests where the tallest trees were smaller than 20 m. However, mature forests on dry sites or at high elevations with limited tree height were not excluded. In addition, we excluded all plots where the water balance was not computable due to unfavorable soil properties, mainly boulder‐rich soils with hollow spaces. Finally, we eliminated several early successional forest plots dominated by birch trees and some heavily managed chestnut groves. The selection procedure resulted in 1,060 seminatural, predominantly late successional mature forest stands. We further included 15 treeless plots in order to extend the drought and the soil aeration gradient. Ten of these plots are located in dry grassy steppes on extremely shallow soils, and the five remaining plots are on marshes. Thus, a total of 1,075 plots were investigated in our study (Fig. 1).

#### 2.2.2 Species data

Species data on the study plots were collected by ~35 authors and partly originate from the Swiss Forest Vegetation Database (Wohlgemuth, 2012). The abundance of the species in the tree layer (mature trees) was assessed during the vegetation survey. Therefore, all the plant species occurring in the herb, shrub, and tree layers in an area ranging from 100 to 500 m^2^ (avg. 200 m^2^) were recorded using the Braun‐Blanquet cover abundance scale (Braun‐Blanquet, 1964; Mueller Dombois & Ellenberg, 1974). As part of the vegetation survey, the height of the forest stand was estimated. The vegetation survey for most of the plots was carried out between 1987 and 2014; however, 34 surveys were completed before 1987. We restricted our assessment to 12 tree species that were present on a sufficient number of plots with regard to species distribution models, for which a minimum of approximately 50 observations is necessary (Coudun & Gegout, 2006). Three species were present in slightly fewer than 50 plots (Table 1). The 12 species belong to two functional groups: broadleaved deciduous (European beech, *Fagus sylvatica*; sycamore, *Acer pseudoplatanus*; European ash, *Fraxinus excelsior*; wych elm, *Ulmus glabra*; cherry tree, *Prunus avium*; pedunculate oak, *Quercus robur*; sessile oak, *Quercus petraea*; downy oak, *Quercus pubescens*; and European hornbeam, *Carpinus betulus),* and needle‐leaved evergreen (Norway spruce, *Picea abies*; silver fir, *Abies alba*; and Scots pine, *Pinus sylvestris*). The three oak species were identified after a revisitation of all plots where oaks occur by jointly considering morphology and molecular‐genetic markers according to Rellstab, Bühler, Graf, Folly, and Gugerli (2016).

**Table 1:** Number and elevation of forest plots with species cover abundance data for the 12 studied tree species. The minimum and maximum elevation of all studied 1,060 mature forest stands across Switzerland was 240 and 2,200 m a.s.l., respectively

Species	Number of plots where present	Elevation (m a.s.l.) where present			Cover abundance (%) where present	
		Minimum	Mean	Maximum	Mean	Maximum
*Picea abies*	684	280	1,005	2,040	33	88
*Abies alba*	410	390	854	1,540	25	88
*Pinus sylvestris*	168	310	820	1,980	24	88
*Fagus sylvatica*	621	340	728	1,490	46	88
*Fraxinus excelsior*	206	240	613	1,360	19	63
*Acer peseudoplatanus*	200	290	811	1,600	17	88
*Ulmus glabra*	61	410	742	1,360	10	63
*Prunus avium*	39	240	515	820	6	38
*Quercus robur*	66	240	528	950	15	63
*Quercus petraea*	97	320	604	1,360	17	88
*Quercus pubescens*	41	330	727	1,360	31	88
*Carpinus betulus*	40	370	506	710	19	88

#### 2.2.3 Environmental data

To estimate the relative importance of the soil and climate variables, and to avoid multicollinearity problems in our analyses, we selected eight predictors according to their suitability for explaining tree species distributions, as described in Walthert et al. (2013). Briefly, an initial set of 87 environmental variables was evaluated and all the variables that were selected had a *r*_s_ < |0.7|. Half of the eight selected predictors were climatic, and the other half were closely related to the soil.

As climate predictors, we selected mean yearly degree‐days with a 5.56°C threshold (DD), mean temperature amplitude between January and July (T‐Cont), mean amount of precipitation from June to August (RR), and mean relative air humidity from June to August (RH; Table 2). Air humidity and temperature amplitude were not tested in Walthert et al. (2013), but both have an impact on plant performance and species distributions (for RH, see Lendzion and Leuschner (2008) and Köcher, Horna, and Leuschner (2012); for T‐Cont, see Jobbagy and Jackson (2000) and Nishimura and Laroque (2011)).

**Table 2:** Environmental predictors at the 1,075 study plots across Switzerland

Category	Name	Class	Description	Min	*Q*_0.25_	Med	*Q*_0.75_	Max	Unit
Climate	DD	Temperature	Mean yearly degree‐days >5.56°C (1981–2010)	575	1,318	1,743	2,025	2,870	°C
	T‐Cont	Temperature	Mean temperature amplitude January/July (1981–2010)	14.6	17.1	18.0	18.4	20.5	°C
	RR	Water	Mean precipitation June to August (1981–2010)	151	342	414	510	792	mm
	RH	Water	Mean relative air humidity June to August (1981–2010)	63.7	71.8	73.0	74.3	80.9	%
Soil	AT/PT	Water	Drought index; mean ratio between actual and potential transpiration June to August (1981–2010)	0.23	0.94	0.99	1.00	1.00	—
	C/N	Nutrients	Mean C/N ratio (C_org_/N_tot_) in 0–10 cm soil depth	7.5	14.5	16.9	20.1	42.5	—
	BS	Nutrients	Mean base saturation in 0–50 cm soil depth	2.7	18.5	85.9	99.6	100.0	%
	W‐level	Soil aeration	Mean depth of the soil water level in the vegetation period	20	200	200	200	200	cm

Climate data for the period 1981–2010 were derived from weather data from the meteorological network of MeteoSwiss. DD values were produced with Daymet (Thornton, Running, & White, 1997). T‐Cont, RR, and RH were calculated using the respective long‐term mean monthly values (1981–2010) provided by Remund, Rihm, and Huguenin‐Landl (2014).

As soil predictors, we selected the drought index (AT/PT) for soil water availability, the C/N ratio (C/N), and base saturation (BS) for soil nutrient availability, and the depth of the water level in the soil (W‐level) for soil aeration (Table 2). The methods for assessing these soil predictors are briefly described in the following text. For a detailed description of these predictors and a justification for their selection, see Appendix S1.

Soil samples were taken from soil profiles, on average 1.2 m deep, according to pedogenetic horizons.

BS in 0–50 cm soil depth: Exchangeable cations (Na, K, Mg, Ca, Mn, Al, Fe) were extracted in a 1 mol/L NH_4_Cl solution and determined by atomic emission spectroscopy (ICP‐AES). Contents of exchangeable protons were determined after extracting the soil in a 1 mol/L KCL solution. The effective cation‐exchange capacity (CEC) was calculated by summing the charge equivalents of exchangeable Na, K, Mg, Ca, Mn, Al, Fe, and H. The base saturation is the percent of exchangeable Na, K, Mg, and Ca of the CEC.

C/N in 0–10 cm soil depth: C/N is the ratio between organic carbon and total nitrogen. Their contents were determined by dry combustion. Possible carbonates were removed by HCl vapor prior to dry combustion.

AT/PT: This index represents the ratio of actual to potential transpiration and corresponds to the average reduction in transpiration due to soil water shortage from June to August in the period 1981–2010. The water balance was modeled on all 1,075 study plots using a coupled mass and heat transfer model for soil–plant–atmosphere systems (Coupmodel). The model was driven by daily weather data and used soil hydraulic parameters that we derived from measured soil properties like texture, density, and stone content. Vegetation was implemented as for a dynamic model forest, and maximum rooting depth was set to 1.5 m. Due to relatively high amounts of precipitation (>900 mm/a) in most parts of Switzerland, drought predominantly occurs on soils with a low storage capacity of plant available water. In the humid suboceanic climate of central Europe, edaphic factors, such as low water holding capacity, are expected to impose more serious constraints on tree water relations than climate (Backes & Leuschner, 2000). Therefore, we assigned the drought index AT/PT to the edaphic predictors. Thus, in our study, water availability is represented by two climatic predictors (RR and RH) and one edaphic predictor (AT/PT).

W‐Level: This variable was used as a proxy for soil oxygen shortage. It is derived from redoximorphic properties and time series of the measured soil water level in the soil pits.

### 2.3 Data analysis

To estimate the relative importance of climate (temperature [DD, T‐Cont] and water [RR, RH]) and soil (water [AT/PT], nutrients [C/N, BS], and aeration [W‐level]), we conducted a variation partitioning analysis (Borcard, Legendre, & Drapeau, 1992; Mood, 1971). We estimated the pure contribution of each predictor set by subtracting the model fit of the opposite set of predictors from the full set of predictors, so that *V*_Predictor subset i_ = *V*_FullModel_–*V*_FullModel without Predictor subset i_. As statistical models, we used generalized linear models (GLM, McCullagh & Nelder, 1989) with logit links, assuming a binomial distribution because the response variables were proportions (i.e., species abundance data). Model fits were evaluated using the adjusted *D* ^2^ (adj.*D* ^2^), following Weisberg (1980). Response curves were derived for each tree species from predictions of GLMs with logit links assuming a binomial distribution. As response variable, we used the cover abundance of mature trees and as predictors the single variables in the linear and quadratic forms. To estimate the GLM performance for each combination of single predictor and species, we estimated with the help of an ANOVA the significance of the difference between the performances of the full model and the model without the specific single predictor. All data were prepared and analyzed using R (R, 2016) and ArcGIS 9.2 (ESRI, 2006).

## 3 RESULTS

### 3.1 Relative importance of climate and soil

The statistical performance (model fits) of the full models including all climatic and edaphic variables varied considerably among tree species. The explained deviance (adj.*D* ^2^) ranged from 0.28 to 0.59 (Table 3). The best model fits (i.e., adj.*D* ^2^ > 0.45) were found for *P. sylvestris*,* F. excelsior*, and *Q. pubescens*. Poor model fits (i.e., adj.*D* ^2^ < 0.30) were found for *P. abies*,* A. pseudoplatanus*, and *A. alba*.

**Table 3:** Pure contributions of individual predictors and of the full set of predictors (full model) for the 12 studied tree species, derived from variable GLMs with cover abundance of mature trees as the response variable and environmental predictors based on data from 1,075 study plots across Switzerland. Numbers indicate the deviance explained by the individual predictors (adj.*D* ^2^ multiplied by 100) and by the full model (adj.*D* ^2^). The direction of the trend (*T*) between the predictor and the response variable is indicated as positive linear “+,” negative linear “−,” positive unimodal “+/−,” or with no clear trend “N.” “Optimum” (Opt.) for DD, RH, AT/PT, and C/N specifies the environmental conditions for which maximal abundance was predicted if the trend was positive unimodal. Boldface indicates adj.*D* ^2^ ≥ 1.5% for individual predictors. For *p*‐values, see (*p*). Abbreviations and units of predictors are explained in Table 2. Note that the sum of the pure contributions of the individual predictors differs from the deviance explained by the full model due to joint contributions

Species	Full model																												
	Climate														Soil														
	Temperature							Water											Nutrients							Soil aeration			Full model
	DD	T	Opt. °C	*p*	T‐Cont	T	*p*	RR	T	*p*	RH	T	Opt. %	*p*	AT/PT	T	Opt.	*p*	C/N	T	Opt.	*p*	BS	T	*p*	W‐Level	T	*p*	
*P. abies*	**5.70**	+/−	1100	.00	0.10	−	.64	−0.10	+	.95	0.00	+		.76	**1.60**	+		.02	**1.70**	+/−	27	.02	0.50	−	.26	0.40	−	.32	0.28
*A. alba*	**5.50**	+/−	1500	.00	0.00	+/−	.86	0.40	+	.40	**3.30**	+/−	76	.00	0.70	+		.28	0.60	+/−	18	.34	0.60	−	.31	0.60	+/−	.29	0.30
*P. sylvestris*	**2.60**	+/−	1900	.06	0.60	+	.47	−0.10	N	.95	1.20	−		.28	0.50	+/−	0.65	.53	**9.50**	+		.00	**2.60**	+	.07	0.50	+	.54	0.45
*F. sylvatica*	**10.10**	+/−	1900	.00	1.20	+/−	.01	0.30	+	.29	0.30	+/−	73	.27	**1.90**	+/−	0.90	.00	1.40	+/−	15	.01	0.50	N	.12	**3.30**	+	.00	0.37
*F. excelsior*	**2.60**	+		.09	0.20	+/−	.77	0.00	−	.90	0.40	−		.62	0.70	−		.48	**8.00**	−		.00	**2.80**	+	.07	1.10	−	.33	0.55
*A. ps.platanus*	−0.10	N		.97	−0.10	−	.98	0.20	+	.73	**1.60**	+/−	75	.24	0.20	+		.74	**7.30**	−		.00	**2.50**	+	.11	1.10	N	.36	0.29
*U. glabra*	**4.80**	+		.35	**3.20**	N	.49	0.20	+	.94	**2.10**	+/−	74	.63	**4.30**	+		.39	**1.50**	−		.71	**7.20**	+	.21	**3.50**	N	.46	0.39
*P. avium*	**4.00**	+		.66	1.40	+	.86	0.70	N	.92	**1.60**	−		.84	0.80	−		.92	**2.90**	−		.74	0.70	N	.92	1.40	N	.86	0.39
*Q. robur*	**8.50**	+		.04	0.80	+/−	.71	1.00	N	.66	0.60	−		.78	**1.90**	+/−	0.80	.47	0.60	−		.78	0.70	N	.73	0.20	N	.90	0.40
*Q. petraea*	**1.90**	+		.35	0.10	+	.88	1.00	N	.57	**1.90**	+/−	68	.34	**4.00**	+/−	0.70	.12	**1.50**	+/−	19	.42	0.40	N	.75	0.50	+	.72	0.30
*Q. pubescens*	0.60	+		.72	1.20	+	.52	1.40	N	.49	**2.50**	−		.27	**9.70**	+/−	0.60	.01	**2.50**	+/−	18	.28	0.20	+	.87	0.20	+	.89	0.59
*C. betulus*	**3.20**	+		.36	0.20	+/−	.92	**4.50**	N	.24	0.90	−		.72	**2.20**	+/−	0.80	.49	**2.20**	−		.48	0.10	N	.92	0.40	N	.86	0.40

Across all 12 tree species, the eight single predictors (in total 96 combinations) had a significant amount of information for the distribution of the tree species in 15 cases (significance level of *p* < .05; Table 3). For most species, *p*‐values and adj.*D* ^2^ were highly correlated.

**Figure 2:**
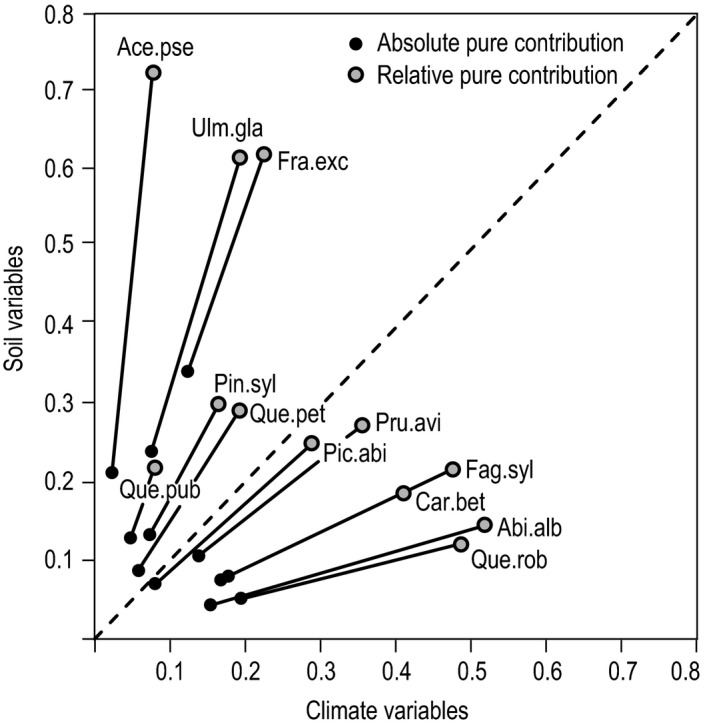
Climate versus soil sensitivity for the 12 studied tree species. Absolute and relative pure contributions of climate and soil variables were derived from grouped variable GLMs with cover abundance of mature trees as the response variable and grouped environmental predictors (four climate and four soil predictors) based on data from 1,075 study plots across Switzerland. Species below the 1:1 line are considered rather sensitive to climate, whereas species above this line are considered rather sensitive to soil variables. The relative pure contributions refer to the full model

For all tree species, models including edaphic and climatic predictors had a better model fit than models including only climatic predictors (Table 4). Further, for all species, the mean pure contribution of the edaphic predictors (adj.*D* ^2^ 0.13 ± 0.09) was larger than the corresponding pure contribution of the climatic predictors (adj.*D* ^2^ 0.11 ± 0.06). However, the pure contributions of the edaphic predictors varied strongly among the tree species: For half of the species (*F. excelsior*,* A. pseudoplatanus*,* U. glabra*,* Q. pubescens*,* P. sylvestris*, and *Q. petraea*), the pure contribution of the edaphic predictors was larger, while for the other half of the species (*A. alba*,* F. sylvatica*,* Q. robur*,* C. betulus*,* P. avium*, and *P. abies*), the pure contribution of the climatic predictors was larger (Fig. 2). The most common tree species in Switzerland, *P. abies*,* A. alba*, and *F. sylvatica* responded rather weakly to soil characteristics.

**Table 4:** Pure and joint contributions of grouped predictors for the 12 studied species, derived from grouped variable GLMs with cover abundance of mature trees as the response variable and environmental predictors based on data from 1,075 study plots across Switzerland. Numbers indicate the deviance explained by the grouped predictors (adj.*D* ^2^). Note that the sum of the pure contributions of the grouped predictors differs from the deviance explained by the full model due to joint contributions

Species	Pure contributions adj.*D* ^2^							Joint contributions adj.*D* ^2^
	Full model	Climate	Soil	Temperature	Water	Nutrients	S. aeration	Climate and soil
*P. abies*	0.28	0.08	0.07	0.06	0.02	0.04	0.00	0.13
*A. alba*	0.30	0.15	0.04	0.09	0.05	0.02	0.01	0.10
*P. sylvestris*	0.45	0.07	0.13	0.04	0.02	0.10	0.01	0.24
*F. sylvatica*	0.37	0.17	0.08	0.14	0.03	0.02	0.03	0.11
*F. excelsior*	0.55	0.12	0.34	0.07	0.01	0.29	0.01	0.09
*A. ps.platanus*	0.29	0.02	0.21	0.00	0.03	0.21	0.01	0.06
*U. glabra*	0.39	0.08	0.24	0.05	0.08	0.21	0.03	0.07
*P. avium*	0.39	0.14	0.11	0.07	0.03	0.08	0.01	0.14
*Q. robur*	0.40	0.19	0.05	0.17	0.02	0.03	0.00	0.15
*Q. petraea*	0.30	0.06	0.09	0.02	0.08	0.04	0.00	0.16
*Q. pubescens*	0.59	0.05	0.13	0.02	0.14	0.03	0.00	0.41
*C. betulus*	0.40	0.16	0.07	0.05	0.03	0.05	0.00	0.16

Across all species, the joint contribution (redundancy) of climate and soil (adj.*D* ^2^ 0.15 ± 0.10; Table 4) was similar to the pure climate (adj.*D* ^2^ 0.11 ± 0.06) and the pure soil contribution (adj.*D* ^2^ 0.13 ± 0.09). Redundancy of soil and climate variables was observed for all species, and the degree of this redundancy, however, varied between species.

### 3.2 Relative importance of temperature, water, nutrients, and soil aeration

**Figure 3:**
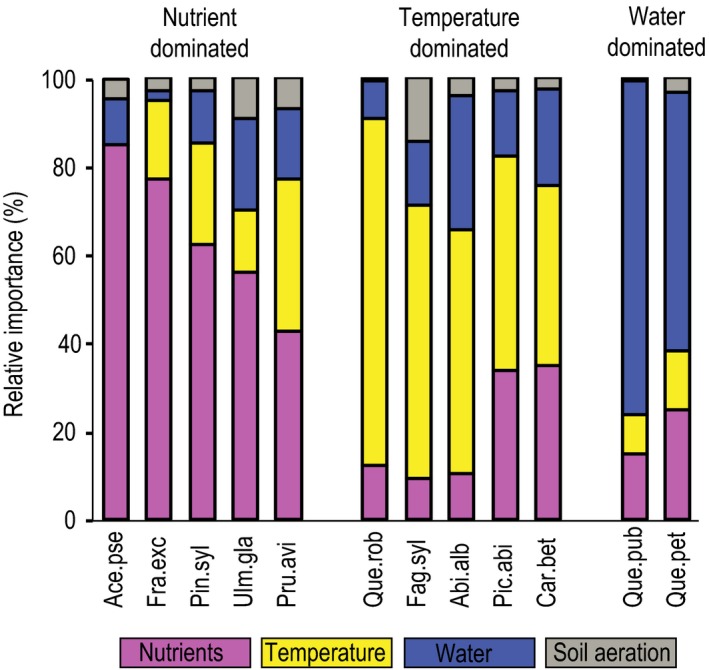
Relative importance of temperature, water, nutrients, and soil aeration in explaining the distribution of the 12 studied tree species. Relative importance is based on pure contributions derived from grouped variable GLMs with cover abundance of mature trees as the response variable and four grouped environmental predictors—temperature, water, nutrients, and soil aeration—based on data from 1,075 study plots across Switzerland. To get relative importance, the pure contributions of the four grouped predictors were converted to relative ones so that the sum of the pure contributions of the four grouped predictors was set to 100% for each species. For pure contributions (adj.*D* ^2^) of the four grouped predictors and of the full models, see Table4

The mean value of the pure contributions of all 12 species showed that the abundance depended mainly on nutrient status (adj.*D* ^2^ 0.09 ± 0.09), followed by temperature (adj.*D* ^2^ 0.07 ± 0.05), water availability (adj.*D* ^2^ 0.04 ± 0.04), and soil aeration (adj.*D* ^2^ 0.01 ± 0.01; Table 4). Based on the individual response patterns, the 12 species were grouped as being mainly sensitive to nutrient status, to temperature or to water availability (Fig. 3).

Species with a predominant response to nutrient status were *A. pseudoplatanus, U. glabra, F. excelsior, P. avium*, and *P. sylvestris* (Fig. 3). Abundance of *A. pseudoplatanus, U. glabra, F. excelsior*, and, to a lesser extent, *P. avium* was positively correlated with nutrient availability. These species preferred soils with high base saturations and low C/N ratios (Table 3). While *P. sylvestris* also responded positively to high base saturations, this species reached the highest abundance on soils with low nitrogen availabilities (high C/N ratios). After nutrient status, temperature, and water availability were of secondary importance for these five species. While *A. pseudoplatanus* responded only marginally to temperature, the four other species required higher temperatures. High air humidity and a good water supply were beneficial for *A. pseudoplatanus* and for *U. glabra*. In contrast, *F. excelsior, P. avium*,* and P. sylvestris* were less water demanding (Table 3; see also Section 3.3.1).

Species that predominantly responded to temperature were *Q. robur, C. betulus, F. sylvatica, A. alba*, and *P. abies* (Fig. 3). While the abundance of *Q. robur* and *C. betulus* increased with temperature, *F. sylvatica, A. alba*, and *P. abies* reached the highest abundance at moderate temperatures; the lowest heat requirement was found for *P. abies* with a maximal abundance at 1,100°C degree‐days, followed by *A. alba* and then *F. sylvatica* with corresponding degree‐days of 1,500°C and 1,900°C, respectively (Table 3). After temperature, water and nutrient status were of secondary importance for these five species. *P. abies, A. alba*, and *F. sylvatica* had higher water requirements than *Q. robur* and *C. betulus* (Table 3; see also Section 3.3.1), with *F. sylvatica* and *A. alba* benefiting most from high air humidity. All five species were rather indifferent to soil nutrient status, most notably with respect to base saturation (Table 3).

A predominant response to water availability was found for *Q. pubescens* and *Q. petraea* (Fig. 3). Both species were most abundant at sites where water supply and air humidity were rather low (Table 3; see also Section 3.3.1). Moreover, both species preferred relatively warm sites. The nutrient status of the soil had only a small influence on these oak species.

### 3.3 Responses of species abundance along drought and soil aeration gradients

#### 3.3.1 Drought sensitivity

Among the three water‐related predictors of our study (RR, RH, and AT/PT), the drought index AT/PT was most able to explain species cover abundances; that is, the pure contribution of this variable was relatively high for the majority of the species with significant values, however, only for *P. abies*,* F. sylvatica*, and *Q. pubescens* (*p* < .05; Table 3).

**Figure 4:**
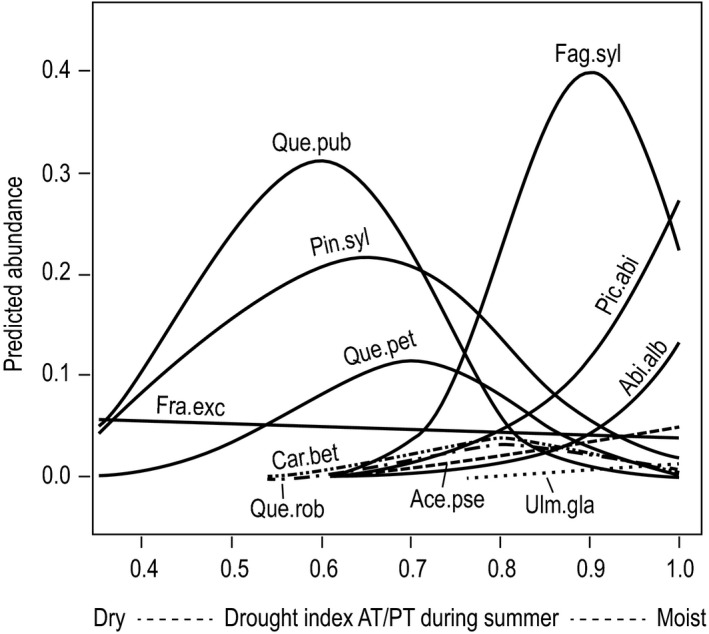
Species sensitivity to drought. Response curves were derived from single variable GLMs with cover abundance of mature trees as the response variable and a drought index (mean AT/PT between June and August, 1981–2010) as predictor based on data from 1,075 study plots across Switzerland. The different species abundance maxima reflect the different mean abundance that species reached on the study plots (Table 1). *Prunus avium* is not illustrated because its response curve runs horizontally at a level of about 0% abundance. If modeled AT/PT is smaller than about 0.3–0.4, forests are not able to persist due to excessive water shortage

The 12 species were arranged in five groups according to their response to AT/PT. The first group, with *P. abies, A. alba*, and *F. sylvatica*, had a maximal abundance under good water supply, that is, at high AT/PT levels, and faded away at AT/PT values of approximately 0.6–0.7 (Fig. 4). The second group, with *A. pseudoplatanus* and *U. glabra*, behaved similar to the first group, but had much lower abundance levels, as is typical for these tree species that rarely dominate in Swiss forests (Table 1). Compared to the species mentioned above, the abundance of *C. betulus* and *Q. robur* peaked on drier sites (AT/PT 0.8). The species of group 4, *Q. pubescens, P. sylvestris*, and *Q. petraea* peaked in abundance in what was clearly the driest range (AT/PT 0.6–0.7) and all extended most toward the dry end of the drought axis. Finally, a unique response was found for *F. excelsior*. This species, though often present on hydromorphic soils, extended into the very dry range of the drought axis.

Based on their response to the drought index AT/PT, that is, the position of the abundance maximum and the shape of the curve toward the dry end of this gradient (Fig. 4), the drought sensitivity of the studied species was ranked as follows: *U. glabra, A. pseudoplatanus, F. sylvatica, P. abies, A. alba > C. betulus, Q. robur > P. avium, Q. petraea, P. sylvestris > F. excelsior, Q. pubescens*.

#### 3.3.2 Sensitivity to limited soil aeration

**Figure 5:**
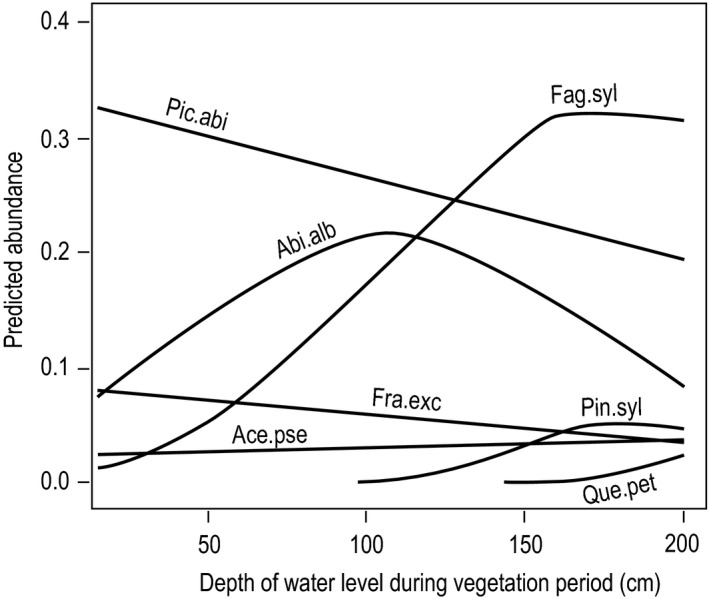
Species sensitivity to limited soil aeration. Response curves were derived from single variable GLMs with cover abundance of mature trees as the response variable and mean depth of a soil water level during the vegetation period (a proxy for soil oxygen availability) as a predictor based on data from 1,075 study plots across Switzerland. The different species abundance maxima reflect the different mean abundance that species reached on the study plots (Table 1). Five species (*Carpinus betulus, Ulmus glabra, Prunus avium, Quercus pubescens*, and *Quercus robur*) are not illustrated because their response curves run horizontally at a level of about 0% abundance

As measured by the pure contributions, soil aeration had little influence on the cover abundance of most tree species (Table 3). For *F. sylvatica*, however, the predictive ability of this variable was quite high (*p* < .001). Our data enabled us to assess the sensitivity to limited soil aeration for five species. Based on the shape of the response curves, we ranked *F. sylvatica* as sensitive and *P. abies*,* A. alba*,* F. excelsior*, and *A. pseudoplatanus* as not sensitive to limited soil aeration (Fig. 5). As we did not have enough observations on strongly hydromorphic soils for *P. sylvestris* and for *Q. petraea*, we did not assess the sensitivity to soil aeration for these species. The same was the case for *C. betulus, U. glabra, P. avium, Q. pubescens*, and *Q. robur*, which had nearly linear and horizontal response curves at low abundance levels and were thus not evaluable.

## 4 DISCUSSION

### 4.1 Relative importance of climate and soil

Variation partitioning revealed that the addition of edaphic variables to the climatic variables improved the model performance for all species. Similar results were already found in earlier research, both for tree species (e.g., Beauregard & de Blois, 2014; Coudun et al., 2006; Piedallu et al., 2013) and for herbaceous species (e.g., Dubuis et al., 2013). Over all species, soil properties were even more important than climate variables in explaining species distribution. Diekmann et al. (2015) found that the distribution of many plant species is strongly driven by soil conditions in regions with low climatic heterogeneity. Our study shows that this seems to be the case not only in climatically homogeneous landscapes but also even in heterogeneous ones like those in Switzerland. Therefore, appropriate soil properties should be included in species distribution models, especially for the species with a strong response to edaphic variables. For the most common species, *P. abies*,* A. alba*, and *F. sylvatica*, however, the inclusion of soil variables is less effective, as their widespread distribution can be interpreted as a result of their relatively low sensitivity to soil characteristics.

Climate is a soil‐forming factor affecting numerous soil processes and soil properties, which could result in a certain degree of redundancy between soil and climate variables in explaining species distribution, as recently hypothesized by Thuiller (2013). A first indication of such a redundancy in our dataset is given by the relatively high correlation between the four climate and the four soil predictors in some cases (Pearson *r* ranging from −0.40 to 0.55 for the 16 combinations). Due to these partly substantial correlations, the redundancy in explaining species distribution, measured as joint contribution of all climate and all soil variables over all species, was of similar magnitude than the pure climate and the pure soil contributions and thus, confirming the hypothesis of Thuiller (2013).

The model performance with the full set of predictors was rather weak for some species. Part of the unexplained deviance could be attributed to biotic factors not included in our models (Meier et al., 2010). Finally, it is noteworthy that the responses were all in accordance with the niche theory (Austin & Smith, 1990), with no multimodal or U‐shaped response curves but only linear trends or unimodal or skewed responses (Table 3 and Fig. 4).

### 4.2 Relative importance of temperature, water, nutrients, and soil aeration

As we expected, the often co‐occurring tree species showed similar response patterns to the four growth factors. Accordingly, the four species *F. excelsior*,* A. pseudoplatanus*,* U. glabra*, and *P. avium* reached highest abundances on nutrient‐rich soils, *Q. petraea* and *Q. pubescens* were most abundant on sites with a low water availability, whereas *Q. robur* and *C. betulus* were restricted to warm sites with frequently changing soil water and soil oxygen availabilities. The three most widespread species, *P. abies*,* A. alba*, and *F. sylvatica*, showed a similar response pattern as well. The characteristic for these species was a low nutrient demand and a relatively high response to temperature.

Across all 12 species, temperature and nutrient availability were most important in shaping species ecological niches. Consistently with the altitudinal distribution of the studied tree species, variation partitioning revealed the different heat demand (degree‐days) of the species (e.g., *F. sylvatica* > *A. alba* > *P. abies*;* Q. robur* > *Q. petraea*). Temperature was a relevant factor not only in our study but also in most other SDM studies (Austin & Van Niel, 2011). Contrary to degree‐days, thermal continentality was a weak predictor for most species in our study. The impact of soil nutrient availability on tree growth is species specific (Lévesque, Walthert, & Weber, 2016). Therefore, it is logical that species differed in their response to nutrients in our study. However, there is an ongoing debate about the degree of feedback between plants and soils. The soil does not only influence species composition, but species also affect soil properties such as nutrient status, mainly by species specific litter input. This bidirectional link between plants and soils is discussed in more detail in Section 4.4. After nutrient status and temperature, water availability was also an important factor for species niche differentiation, much more so than soil aeration. Among the three predictors that represented water availability, the drought index AT/PT was by far most important (see Section 4.3.1), followed by air humidity and precipitation, which was a weak predictor for most species. Species that preferred oceanic climate with high mean air humidity were *A. alba*,* A. pseudoplatanus* and *U. glabra*, whereas *Q. pubescens*,* P. sylvestris,* and *P. avium* were most abundant in continental regions with often relatively low air humidity. The importance of soil aeration could be evaluated only for a minority of the studied species (see Section 4.3.2).

### 4.3 Responses of species abundance along drought and soil aeration gradients

Future summers will probably be warmer and drier, while precipitation in winter and spring may be enhanced (Lindner et al., 2014). Increasing summer droughts and decreasing oxygen availability due to waterlogging during winter and spring may adversely affect the growth and competitive ability of sensitive tree species on many forest sites (Kreuzwieser & Gessler, 2010). With these future prospects, it is important to know the sensitivity of species to drought and to soil oxygen shortage.

#### 4.3.1 Drought

Almost every physiological process in plants is affected directly or indirectly by water supply (Kramer & Boyer, 1995). In the past, the drought tolerance of many tree species has been thoroughly explored, mainly on the basis of case studies. However, it is unclear how well these local assessments reflect the large‐scale situation in forest ecosystems. Moreover, previous studies have often focused on juvenile plants, although juvenile and mature plants may differ in physiology (Kolb & Matyssek, 2001; Ryan & Yoder, 1997). Furthermore, growth conditions simulated in experiments diverge from the multifactorial field conditions (Kolb & Matyssek, 2001).

Based on large‐scale inventory data from about 1,000 mature forest stands, we showed that the 12 studied tree species varied greatly in their drought sensitivity, that is, in their response to the summer drought index AT/PT. Our results are in good qualitative agreement with comparable research exploring species drought sensitivity, such as (1) case studies investigating physiological characteristics of mature trees in Central European forest stands (e.g., Zweifel, Rigling, & Dobbertin, 2009) or juvenile trees in greenhouse experiments (e.g., Arend, Brem, Kuster, & Gunthardt‐Goerg, 2013); (2) a Swiss case study measuring the canopy foliage temperature of mature trees with infrared thermography (Scherrer, Bader, & Körner, 2011); (3) case studies on growth dynamics of mature trees in old‐growth European forests using tree rings (e.g., Cavin, Mountford, Peterken, & Jump, 2013); and (4) large‐scale tree regeneration and mortality assessments in mature Swiss forests (Rigling et al., 2013). However, the comparison between our study and the above‐mentioned drought‐oriented research is not exhaustive. First, comparable data were hardly available for some of the 12 studied species, especially for *U. glabra, P. avium*, and *C. betulus*. Second, with only three species on average, the number of simultaneously investigated species was rather low in most of these previous studies focused on drought. Nevertheless, our study based on large‐scale inventory data indicates that species drought sensitivities, as assessed in case studies and experiments, are well reflected in species cover abundance in mature forest stands across Switzerland. Moreover, species' response patterns to the drought index AT/PT indicated that drought was an important determinant of the lower range boundary for some species, as hypothesized by Normand et al. (2009) and Anderegg and HilleRisLambers (2016).

#### 4.3.2 Soil aeration

Trees are aerobic organisms that depend on a steady supply of oxygen to all living cells. A shortage of oxygen therefore disturbs the plant metabolism. During waterlogging, oxygen diffusion into the soil and oxygen supply to the roots are reduced (Kozlowski, 1986). Many energy‐consuming processes, including shoot and root growth, are slowed down to overcome the energy crisis caused by waterlogging (Kreuzwieser & Rennenberg, 2014). Current knowledge on the responses of European tree species to waterlogging and oxygen shortage has been acquired mainly in case studies based on (1) physiological, morphological, and growth reactions of seedlings under controlled conditions (e.g., Dreyer, 1994; Schmull & Thomas, 2000), (2) aboveground damage to mature trees in open land (review from Kreuzwieser & Rennenberg, 2014), and (3) time until plant dieback occurred (review from Niinemets & Valladares, 2006). In general, species cover abundance on the approximately 1,000 study plots across Switzerland reflected species sensitivity ratings from these case studies. However, as already observed for drought sensitivity, comparisons between published research and our study suffered from scarce data availability. Moreover, we could compare only five species (*F. sylvatica, P. abies, A. alba, F. excelsior*, and *A. pseudoplatanus*), as our sensitivity assessment was feasible only for these species. Discrepancies between our and published results were only found in the case of Niinemets and Valladares (2006), who stated that *A. alba, P. abies*, and *A. pseudoplatanus* are very intolerant to waterlogging. In our study, in contrast, these species reached a relatively high abundance on many strongly hydromorphic soils. The ability of *P. abies* to adapt to waterlogging is remarkable. In strongly hydromorphic soils, this species develops a shallow rooting system to avoid deeper anaerobic horizons.

### 4.4 Feedback between plants and soils

The postulated relevance of soil variables for tree species distribution may arise from circular reasoning. Vegetation is a soil‐forming factor and thus modifies different soil characteristics mainly in the topsoil (Augusto, Dupouey, & Ranger, 2003). Even though such topsoil modifications may be considerable and may occur relatively fast within the lifetime of a tree species (Cools, Vesterdal, De Vos, Vanguelova, & Hansen, 2014; Vesterdal, Schmidt, Callesen, Nilsson, & Gundersen, 2008), the influence of tree species on soil should not be overrated as geological characteristics and other site factors seem to influence soil properties at least as much as tree species do (Augusto et al., 2003; Prescott, 2002). Therefore, the apparent circular reasoning in our study may be only weak meaning that soil data may well be used as variables for analyzing tree species distribution.

### 4.5 Importance of forest management

Silvicultural interventions have the potential to smear the ecological niches of tree species. Past forest management probably changed tree species composition on many plots of our study, although a near‐natural tree species composition was a precondition for the selection of most plots. In our study, the highest influence of past forest management is expected for *P. abies* in lowlands where this species has been fostered by silvicultural activities at many places. However, around half of the 684 plots with occurrence of *P. abies* are located above 1,000 m a.s.l. where the abundance of this species is naturally important on many forest sites due to harsh climate. Thus, careful plot selection and many high altitudinal plots might have dampened the spurious impact of forest management on the data, the results, and the study conclusions.

## 5 CONCLUSIONS

The edaphic predictors used in our study improved the statistical model performance (i.e., the model fit) for all studied tree species. Moreover, with two of our edaphic predictors, which quantified drought and soil oxygen shortage, we were able to show that species ecological requirements assessed in local field studies and experiments are generally well reflected in forests on larger scales across Switzerland. These findings underline the ecophysiological relevance of the edaphic predictors used in our study.

Our thoroughly data‐driven assessment is expected to deliver an objective insight into species ecological niches. It may complement the rather qualitative knowledge provided by the system of indicator values. Our results may be used as basic information for forest management and habitat protection in general and specifically for forest conversion under a changing climate.

## CONFLICT OF INTEREST

None declared.

## AUTHOR CONTRIBUTIONS

L.W. conceived the ideas, provided the data, and led the writing, and E.S.M. analyzed the data and assisted in writing.

## DATA ACCESSIBILITY

The data of this study are archived in the soil data base of the Swiss Federal Institute for Forest, Snow and Landscape Research WSL and are available on request. Contact person: Lorenz Walthert (lorenz.walthert@wsl.ch).
