# Supplementary material for: Tree species distribution in temperate forests is more influenced by soil than by climate
Source: Ecol Evol. 2017 Oct 11;7(22):9473–84. doi: 10.1002/ece3.3436 (PMC5696420; doi:10.1002/ece3.3436)
Supplement: Supplementary file 1 [file ECE3-7-9473-s001.pdf]

SUPPORTING INFORMATION

Tree species distribution in temperate forests is more influenced by soil than by climate

L. Walthert and E. S. Meier

Appendix S1

Detailed description of soil sampling and soil analyses

Soil description and soil sampling: Soil pits were located within or very near the vegetation plots. Soil pits, on average 1.2 m deep, were described morphologically according to pedogenetic horizons that were distinguished by criteria such as colour, structure, volumetric stone content and the occurrence of redoximorphic features (Walthert *et al.*, 2004). Rooting depth and possible barriers for roots, like compact parent rock or permanently anaerobic horizons, were recorded based on visual observations in all soil pits. For chemical analyses, an average of six soil samples were taken from each soil profile according to pedogenetic horizons. Volumetric samples were collected in a subset of the soil pits in order to determine the density of the soil. These samples were taken with steel cylinders 1000 cm^3 in volume and in stony soils by using quartz sand. If a forest floor was present, the organic horizons were sampled as well. Thus, organic layers were fully considered in our study.

Sample preparation and storage: Soil samples for chemical analyses were dried at 40–60 °C until constant weight and sieved to 2 mm. An aliquot of each sample was

ground for 3 min using a vibrating ball mill (Retsch MM2000) with zircon-grinding tools. All samples were stored in the dark at room temperature and at an atmospheric humidity between 40 % and 50 %. Volumetric samples were dried at 105 °C for 48 h.

Soil physical properties: Soil texture and density data are required for modelling the water balance. For the mineral horizons of around 750 soil pits, particle sizes analysed gravimetrically according to Gee and Bauder (1986) were available. For the remaining soil samples, we used the field estimates based on ten texture classes from Walthert *et al.* (2004). For a subset of soil profiles, the densities of the soil (including stones > 2 mm) and of the fine earth (all particles < 2 mm) were determined by weighing oven-dried volumetric soil samples and sieving the samples in a water bath to quantify the weight of stones. The volume of stones was calculated by assuming a density of 2.65 Mg/m^3 for stones. With the density data from 168 forest soils, Nussbaum *et al.* (2016) developed a pedotransfer function (PTF) that can be used to estimate the density of the fine earth from easily ascertainable site and soil properties. We used this PTF to estimate the density for all mineral horizons of the 1075 studied soil pits. The values for densities of organic horizons in the forest floor were taken from Moeri (2007) as follows: OF: 0.15 Mg/m^3, OH: 0.20 Mg/m^3.

Base saturation (BS): Exchangeable cations were extracted (in triplicate) from the 2-mm-sieved soil in an unbuffered solution of 1 M NH_4Cl for 1 h on an end-over-end shaker using a soil-to-extract ratio of 1:10. The element concentrations in the extracts were determined by ICPAES (Optima 3000, Perkin–Elmer). Contents of exchangeable protons were calculated as the difference between the total and the Al-induced exchangeable acidity, as determined (in duplicate) by the KCl method (Thomas, 1982). This method was applied only to soil samples with a pH ($CaCl_2$) <

6.5. In samples with a higher pH, we assumed the quantities of exchangeable protons were negligible. The effective cation-exchange capacity (CEC) was calculated by summing the charge equivalents of exchangeable Na, K, Mg, Ca, Mn, Al, Fe and H. The base saturation was defined as the percent of exchangeable Na, K, Mg, and Ca of the CEC.

We used BS instead of pH as an indicator for the alkali-acidity status of the soil and for nutrient availability. This was done because, compared with pH, BS had a much stronger correlation with plant-available nutrient cations (Ca-, Mg-, and K-contents) and with potentially toxic Al-contents in the frequently occurring acidic pH range (pH $CaCl_2$ 2.6–5.0, data not shown).

C/N-ratio (C/N): The total carbon and total nitrogen contents were determined by dry combustion. This involved weighing 25 mg of ground samples into tin capsules (in duplicate), followed by combustion with a CN analyser NC 2500 (CE Instruments, Italy). For samples with a pH ($CaCl_2$) < 6.0, we assumed that the organic carbon content was equal to the total carbon content. All samples with a pH > 6.0 were assumed to potentially contain carbonates. The organic carbon content of these samples was determined following Walthert *et al.* (2010). Briefly, prior to dry combustion, all carbonates were removed by fumigating the samples with HCl vapour. The C/N-ratio is the quotient of organic carbon and total nitrogen and is considered to be a robust indicator of nitrogen availability in the soil (Andrianarisoa *et al.*, 2009).

Calculation of C/N and BS for fixed soil depths: According to Walthert *et al.* (2013), the topsoil influences the cover abundance of tree species more than the subsoil does, and the soil depths most suitable for plant-soil studies like ours was

found to be 0-10 cm for C/N and 0-50 cm for BS. Thus, we converted the element contents from the pedogenetic horizons to these recommended soil depths before calculating C/N and BS. In doing so, the contribution of the pedogenetic horizons involved in the mean element content of a fixed soil depth was proportional to the amount of fine earth of the pedogenetic horizons in that depth. The amount (weight) of fine earth (<2 mm) in each pedogenetic horizon was calculated from the three parameters: horizon thickness, volumetric stone content and density of the fine earth.

Drought index (AT/PT): The water balance on the 1075 study plots was modelled in daily time steps for the period 1981–2010 to estimate the risk of drought using the drought index AT/PT, i.e. the ratio of actual to potential transpiration. In our study, AT/PT corresponded to the average reduction in transpiration due to soil water shortages from June to August during the period 1981–2010. The water balance was modelled with the Coupmodel (Jansson & Karlberg, 2011), a mass and heat transfer model for soil-plant-atmosphere systems. A detailed description of all modelling steps including model calibration and validation was provided by Walthert *et al.* (2015). We calibrated the model with long-term data on forest stand interception from 12 Swiss Level II plots (Thimonier *et al.*, 2005) of the Swiss Long-term Forest Ecosystem Research programme LWF. Soil water potential was calibrated based on long-term data from 11 Swiss Level II plots (Graf Pannatier *et al.*, 2011) and 13 other forest plots located across Switzerland (Richard *et al.*, 1978-1987). Soil water potential was validated with long-term data from 9 forest stands (data not published) not used for model calibration.

The required soil hydraulic parameters (van Genuchten parameters) were estimated according to Teepe *et al.* (2003) from soil texture and soil density. We further considered volumetric stone content and soil hydromorphic properties in the

modelling process. Daily weather data (air temperature, precipitation, radiation and relative humidity) were interpolated for our study plots based on data of the meteorological network of MeteoSwiss (Remund *et al.*, 2014). Vegetation data was implemented as for a model-forest with properties, e.g. leaf area index (LAI) and its development during the year, based on investigations in Swiss forests (e.g. Schleppi *et al.*, 2011). Rooting depth extended to a barrier for roots in the soil pit or to a maximum depth of 1.5 m if such a barrier was not present.

The validation of modelled soil water potential time series data yielded satisfactory results (Walthert *et al.*, 2015). Due to the general lack of appropriate data, it was not possible to validate the modelled time series of AT/PT. However, the average AT/PT from 1981–2010 during summer and the height of the forest stand were quite strongly correlated in 507 mature forests below 1000 m a.s.l., indicating that soil water availability had a considerable influence on tree growth. The climatic water balance and the stand height showed a much weaker correlation in the same period. These findings agree with Piedallu *et al.* (2016), who stated that simple climatic water indices can be inadequate for representing the soil water available for plants, and furthermore demonstrate the need to consider the soil in water balance estimations.

Soil aeration (W-Level): We used the mean depth of the water level in the soil during the vegetation period as a proxy for soil aeration intensity, i.e. soil oxygen shortage. The oxygen availability in the waterlogged soil below the water level is usually strongly reduced. The water level in all of the 1075 studied soils, however, was not measured but derived in the following three steps. First, the intensity of soil hydromorphy was classified for all soils, following Walthert *et al.* (2004), based on the redoximorphic features assessed in the soil profiles. Six intensity classes are available for soils with stagnic properties (inhibited drainage), and five classes are

available for soils with gleyic properties (groundwater). Second, data on the relationship between the classified intensities of soil hydromorphy and the depth of the water level in the soil were obtained using long-term data on soil water potential from 11 soils showing different intensities of soil hydromorphy (Richard *et al.*, 1978-1987; Graf Pannatier *et al.*, 2011). In doing so, we reconstructed the course of the water level in the soil during the year in each of these soil profiles and evaluated the courses for the different classes of soil hydromorphy (Walthert *et al.*, 2015). The mean depth of the water level during the vegetation period depended on the intensity of soil hydromorphy, with values ranging from 65 to 200 cm depth in stagnic soils and from 20 to 200 cm in gleyic soils. Third, we assigned the appropriate depth of the water level to each of the 1075 soil profiles based on the classified intensity of soil hydromorphy. In soils without any redoximorphic features, the water level was set to a depth of 200 cm.

Oxygen shortage in the soil has negative impacts on tree metabolism (Kreuzwieser & Rennenberg, 2014), especially for sensitive species. The inclusion of data related to soil aeration could be important in our study because temporary or permanent waterlogging is widespread in many forest soils of the Swiss plateau and in the northern Alps. As we have no sites in our dataset where flooding occurs, waterlogging means that only the soil is saturated with water and that there is no water standing above the soil level.

References

Andrianarisoa, K.S., Zeller, B., Dupouey, J.L. & Dambrine, E. (2009) Comparing indicators of N status of 50 beech stands (Fagus sylvatica L.) in northeastern France. *Forest Ecology and Management*, **257**, 2241-2253.

Gee, G. & Bauder, J. (1986) Particle-size analysis. *Methods of soil analysis, Part 1: Physical and Mineralogical Methods (2nd Edition)* (ed. by A. Klute), pp. 383-411. Agronomy Monograph No 9, Madison WI.

Graf Pannatier, E., Thimonier, A., Schmitt, M., Walthert, L. & Waldner, P. (2011) A decade of monitoring at Swiss Long-Term Forest Ecosystem Research (LWF) sites: can we observe trends in atmospheric acid deposition and in soil solution acidity? *Environmental Monitoring and Assessment*, **174**, 3-30.

Jansson, P.-E. & Karlberg, L. (2011) *CoupModel: Coupled heat and mass transfer model for soil-plant-atmosphere systems. Technical manual for the CoupModel.*, Stockholm, available online: https://www.coupmodel.com/documentation.

Kreuzwieser, J. & Rennenberg, H. (2014) Molecular and physiological responses of trees to waterlogging stress. *Plant, Cell & Environment*, **37**, 2245-2259.

Moeri, A.C. (2007) *Kohlenstoffvorräte in Schweizer Waldböden mit besonderer Berücksichtigung der organischen Auflage*. Geogr. Inst. Uni Zürich, Eidgenössischen Forschungsanstalt für Wald, Schnee und Landschaft (WSL).

Nussbaum, M., Papritz, A., Zimmermann, S. & Walthert, L. (2016) Pedotransfer function to predict density of forest soils in Switzerland. *Journal of Plant Nutrition and Soil Science*, **179**, 321-326.

Piedallu, C., Gegout, J.C., Lebourgeois, F. & Seynave, I. (2016) Soil aeration, water deficit, nitrogen availability, acidity and temperature all contribute to shaping tree species distribution in temperate forests. *Journal of Vegetation Science*, **27**, 387-399.

Remund, J., Rihm, B. & Huguenin-Landl, B. (2014) *Klimadaten für die Waldmodellierung für das 20. und 21. Jahrhundert. Schlussbericht des Projektes im Forschungsprogramm Wald und Klimawandel.* Eidg.

Forschungsanstalt für Wald, Schnee und Landschaft WSL. avaiable online: http://dx.doi.org/10.3929/ethz-a-010693673.

Richard, F., Lüscher, P. & Strobel, T. (1978-1987) *Physikalische Eigenschaften von Böden der Schweiz (Lokalformen)*. Eidg. Anstalt für das forstliche Versuchswesen und ETH Zürich Fachbereich Bodenphysik. 4 Bände.

Schleppi, P., Thimonier, A. & Walthert, L. (2011) Estimating leaf area index of mature temperate forests using regressions on site and vegetation data. *Forest Ecology and Management*, **261**, 601-610.

Teepe, R., Dilling, H. & Beese, F. (2003) Estimating water retention curves of forest soils from soil texture and bulk density. *Journal of Plant Nutrition and Soil Science*, **166**, 111-119.

Thimonier, A., Schmitt, M., Waldner, P. & Rihm, B. (2005) Atmospheric deposition on Swiss Long-term Forest Ecosystem Research (LWF) plots. *Environmental Monitoring and Assessment*, **104**, 81-118.

Thomas, G.W. (1982) Exchangeable cations. *Methods of Soil Analysis, Part 2: Chemical and Microbiological Properties (2nd Edition)* (ed. by A.L. Page), pp. 159-165. Agronomy Monographs No 9, Madison, WI.

Walthert, L., Graf Pannatier, E. & Meier, E.S. (2013) Shortage of nutrients and excess of toxic elements in soils limit the distribution of soil-sensitive tree species in temperate forests. *Forest Ecology and Management*, **297**, 94-107.

Walthert, L., Zimmermann, S., Blaser, P., Luster, J. & Lüscher, P. (2004) *Waldböden der Schweiz: Band 1. Grundlagen und Region Jura*. Hep Verlag, Bern.

Walthert, L., Graf, U., Kammer, A., Luster, J., Pezzotta, D., Zimmermann, S. & Hagedorn, F. (2010) Determination of organic and inorganic carbon, delta C-13, and nitrogen in soils containing carbonates after acid fumigation with HCl. *Journal of Plant Nutrition and Soil Science*, **173**, 207-216.

Walthert, L., Scherler, M., Stähli, M., Huber, M., Baltensweiler, A., Ramirez-Lopez, L. & Papritz, A.J. (2015) *Böden und Wasserhaushalt von Wäldern und Waldstandorten der Schweiz unter heutigem und zukünftigem Klima (BOWA-CH). Schlussbericht des Projektes. In German with English summaries*. Eidg. Forschungsanstalt für Wald, Schnee und Landschaft WSL. available online: http://dx.doi.org/10.3929/ethz-a-010658682.
